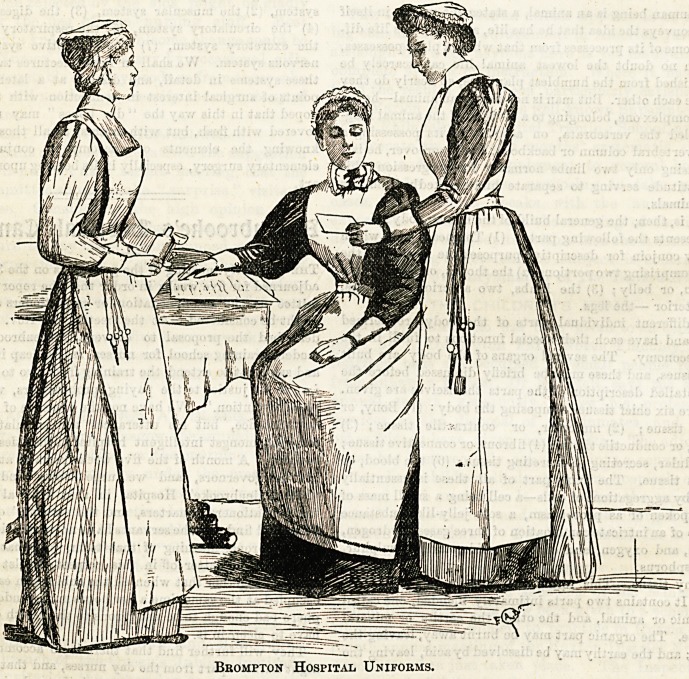# The Hospital Nursing Supplement

**Published:** 1895-01-12

**Authors:** 


"The Hospital" Nursing Mirror.

Being the Extra Nursing Supplement of "The Hospital" Newspaper.

[Contributions for this Supplement should be addressed to the Editor, The Hospital, 428, Strand, London, W.C., and should have the word "Nursing" plainly written in left-hand top corner of the envelope.]

### News from the Nursing World.

### ROYAL GIFTS.

The New Year's gifts of her Majesty were distributed in the riding school at Windsor Castle, the Mayor, the Dean of Windsor, and many other visitors being present on the occasion. The Queen's bounty takes the form of beef, in quantities varying from three to seven pounds, and the recipients are the poor of the Clewer, Holy Trinity, and Windsor parishes. Coals also form part of the New Year's gift, and are delivered at the people's homes in from one to three hundredweights. The Royal Clothing Club is another of the local charities in which her Majesty takes personal interest, and to which she presents £100 annually.

### PRINCESS LOUISE AT CHELSEA.

When the Victoria Hospital for Sick Children was quite a small establishment, with no Nurses' Home, nor indeed with any of the supplementary buildings of which it is now possessed, it secured the deep sympathy and helpful interest of Her Royal Highness the Princess Louise. Few people noted the arrival at various times of two ladies who often drove quietly up and made careful inspection of every department, entering into details of management, and observing the routine of the in-and-out-patient departments with evident knowledge of the subject. Sometimes a small patient, better informed than the others, would point out one of the ladies as "the daughter of our very own Queen, nurse says!" On Friday last the children were the recipients of personal service from their Royal friend and patron the Princess, and the Marquis of Lorne kindly consented to be present at the Annual Christmas Entertainment, when they assisted energetically in distributing the gifts with which the trees were laden and surrounded.

### BEWARE OF SPURIOUS CO-OPERATIONS AND HOMES.

We have recently felt it our duty, in the interest of nurses, in consequence of certain apparently well-founded complaints which have reached us, to cause inquiries to be made as to the management of a so-called Nurses' Co-operation and Home in a northern city. The inquiry has revealed that after paying travelling expenses and charges for board and lodging in advance, on the faith of representations that remunerative work would be provided, nurses have found themselves compelled for the sake of their own reputation and to escape from discomfort, insult, assault, extortion, and other evils, to leave the home, some within a few days, some after longer residence, without obtaining any work. There are doubtless many very respectable private adventure nursing homes in our large towns, which confer valuable benefits on nurses, but we earnestly warn all who may think of joining such a home to make close and careful inquiries about it and its proprietor or manager beforehand, and to ascertain that it is not either the establishment or resort of drunken, profligate, or discredited proprietors or nurses, or an institution bringing profit and benefit to its proprietor alone, and that by means of misrepresentation, intimidation, and extortion.

### WHO PAYS?

"Why do you refuse to take a week's holiday, Sister, when you really need a change, and Matron is anxious you should have it?" asked the Charge Nurse, knowing better than anybody else how tired Sister had looked of late. "To tell the truth, Nurse, the Christmas expenses have been very heavy, and I can't afford to go away till next quarter's salary is due." "Well," commented the Nurse, "I don't think it's fair that expense as well as labour should fall on the workers. Yet, of course, the patients must have a good time!" Whilst gifts and contributions flow freely into some institutions, in others sisters and charge nurses appear to provide the bulk of the presents for the patients, decorations for the ward, and the necessary "light" refreshments. It is pleasant to find that pianos for the Christmas entertainments were lent to Guy's Hospital by Messrs. Broadwood, Brinsmead, Erard, Blüthner, D'Almaine, Steinway, Stiles, and Chappell, and that Messrs. Doulton annually loan some beautiful pottery to adorn the wards at St. Thomas's Hospital. Whilst the visiting surgeons and physicians hold varying views as to obligations to contribute to festivities, residents and students seem to second the nursing staff's efforts to the utmost extent of their abilities. It is to he hoped that in the future the purses of nurses and sisters will no longer be annually taxed!

### NURSES AT OXFORD.

It is proposed by the promoters of the memorial to Sir Henry Acland to secure improved accommodation for the nurses and patients at the Sarah Acland Memorial Home at Oxford, the present houses being found inadequate to meet the demands made upon their resources. A trained superintendent supervises the Home, and has under her five district and twenty-eight private nurses, besides a home for paying patients. So far the expenditure has, according to the reports, exceeded the receipts, but Miss Acland, as treasurer, has kindly collected subscriptions to clear off the debt. In considering the scheme of the proposed memorial the subscribers will doubtless question whether the present system does not admit of modification. A combination of the charity of nursing the sick poor with the business of supplying private nurses is not often satisfactory, the difficulty being so to apportion the expenses as to ensure that the payment for the nurses shall not be used to lighten the call upon the charity.

### UNACCEPTABLE BECAUSE UNFAMILIAR.

"I hope you are enjoying your dinner to-day," remarked the urbane visitor. "Do you know, my man, that you are being treated to some pheasants from my own place in the country?" The patient looked incredulous. "Is this really what yer calls a pheasant, sir?" "Certainly it is. What did you take it for?" "Well," said the man, "if you wants to know, I took it for a bad fowl, and, thanking you all the same, sir, I'd be glad to stick to my reg'lar diet while I'm 'ere. I knows mutton and beef, aye, and a rabbit when I sees 'em, but I don't 'old with fancy dishes." The visitor discussed the patient's want of taste with the matron, but was shocked at her suggestion that game rejected by the sick might be gladly accepted for the use of the nursing staff. Most superintendents know what a welcome change could be made in the Nurses' Home dietary by occasional gifts of animal or vegetable country produce from those who condemn but will not help to rectify the monotony of institution fare.

### WEST COUNTRY GUARDIANS.

The Guardians at Helston negatived the suggestion of one of their number at a recent meeting, that the Visiting Committee should make "surprise" visits to the workhouse. Considering the high opinion they hold of their officials it is somewhat strange that they cannot venture to pay them unannounced calls! A document purporting to be a "reply" to the report of the Local Government Board Inspector was laid before the Guardians, who appeared to esteem its flat contradictions of some of the statements perfectly satisfactory. An average of five hours a week out of the "house" is hardly sufficient recreation for the nurse, who has, we believe, no qualified substitute to take her place in her absence.

### NEW YEAR'S GREETINGS AT GLASGOW.

At the Royal Infirmary, Glasgow, the annual gathering of the nurses took place on the morning of New Year's Day, in the dispensary, under the presidency of the Lord Provost. It was the twenty-seventh anniversary on which the meeting had been held, and the hearty good wishes of the assembled guests accompanied the Lord Provost's kindly "God-speed" to the nurses. An anthem was sung by the cathedral choir, and several speeches followed.

### PROGRESS IN CORK.

Charleville has every prospect of soon possessing a trained district nurse of its own, and Miss Dunne, of the Irish branch of the Queen's Jubilee Institute, accepted an invitation the other day to speak in the Dispensary Hall on the subject. She gave an excellent outline of the organisation in Liverpool, since followed in other places, for giving skilled nursing to the poor in their own homes. At the close of her particularly interesting address Miss Dunne answered a number of questions put to her by those present, and in the evening she spoke at a meeting of ladies. A committee was subsequently appointed to arrange the details of the scheme, which it was unanimously agreed would be highly beneficial to the neighbourhood.

### VERY YOUNG MIDWIVES.

In Paris the study of midwifery appears to attract such young pupils, that at some of the maternity hospitals a certain number have obtained admission when under nineteen years of age. It has now been officially notified that even if they enter the schools earlier, "the scholastic curriculum" will in future only be reckoned after the pupils are nineteen.

### THE LEGION OF HONOUR.

The nursing in the Naval Hospital at Brest is done by the Order of La Sagesse, whose Superior, Sister Agnes, has recently received the "Legion of Honour." She is said to be the twenty-ninth professed Sister who has been thus decorated. The first to receive the Order was Sister Martha (Madame Biget), on whom it was bestowed by the Emperor Napoleon in 1815.

### MENTAL NURSES IN U.S.A.

The training of mental nurses appears to be receiving considerable encouragement in America just now although but a few asylums have as yet inaugurated a complete system of instruction. Where it has been tried the results have been particularly satisfactory. Miss Marion E. Smith, the well-known Superintendent of the Philadelphia Hospital Training School, appears to advocate six or twelve months' systematic asylum work as a desirable supplement to general hospital training for all nurses proposing to take private cases. Miss Smith speaks with the authority of experience gained through the nursing of the insane department of the hospital with which she is connected. An interesting article on the subject is contributed by her to the January number of *The Trained Nurse.*

### THE CHILDREN'S HOME.

This pleasant title has been given to an establishment at 1, Torrington Park, North Finchley, where fourteen beds are occupied by small people rescued from unhappy homes. Dr. Swindell, says the second annual report, generously places his professional services at the disposal of the inmates, and many other friends have given useful gifts. The adjoining house has been recently converted into a convalescent home where children discharged from hospitals can be received and treated. It is under the care of a trained nurse-matron, and the terms charged for the children are moderate. Funds are asked for to carry on the work.

### SHORT ITEMS.

It is reported that the sanction of the Minister of Public Instruction has now been given to a scheme for allowing medical women to practice in Russia.—The fourth annual meeting of the Buxton District Nursing Association has just taken place. The Inspector of the Queen's Jubilee Institute, with which the association is affiliated, reports well of the work done.—An appeal is being made at Teignmouth for funds to establish a trained nurse for the benefit of the sick poor in the town.—A pleasant entertainment was given on New Year's Day in aid of the Mansfield and District Nursing Fund.—Miss Mary Florence Elsdon, for ten years a nurse at the Poplar and Stepney Sick Asylum, was married last month to Mr. H. Wright, of the Press Association.—The late gales damaged the Hospital Ship "Albert" and the Mission Ship "Thomas Gray," both belonging to the M.D.S.F., and they are undergoing repairs at Yarmouth.—An entertainment was given in aid of the funds of the Loswithiel Nursing Association on 2nd inst.

### Elementary Anatomy and Surgery for Nurses.

By W. McAdam Eccles, M.B., B.S., F.R.C.S., Lecturer to Nurses, West London Hospital.

### I.—INTRODUCTORY.

"What is man?" is a question which has been handed down through ages, and many a philosopher and scientist has sought to answer it. To-day it still remains to a great extent an unsolved problem, although anatomists and physiologists have largely increased our knowledge of the structure and functions of his animal body. It is the purport of the following course of lectures to review briefly but as clearly as possible the most important facts in the anatomy of man, and while his anatomy is being dealt with to describe the chief surgical diseases and accidents to which his tissues and organs are liable.

The human being is an animal, a statement which in itself merely conveys the idea that he has life, and that this life differs in some of its processes from that which a plant possesses, although no doubt the lowest animal life can scarcely be distinguished from the humblest plant life, so nearly do they resemble each other. But man is not simply an animal—he is a highly complex one, belonging to a division of the animal kingdom called the vertebrata, on account of its possessing a typical vertebral column or backbone, and, moreover, he is a biped, using only two limbs normally for progression; his erect attitude serving to separate him markedly from the lower animals.

What is, then, the general build of the human body?

It presents the following parts: (1) The head, with which one may conjoin for descriptive purposes the neck; (2) the trunk, comprising two portions (*a*) the thorax, or chest, (*b*) the abdomen, or belly; (3) the limbs, two anterior—the arms, two posterior—the legs.

The different individual parts of the body are termed organs, and have each their special functions to fulfil in the animal economy. The several organs of the body are built up of tissues, and these must be briefly discussed before the more detailed description of the parts themselves are given. There are six chief tissues composing the body: (1) Bony, or osseous tissue; (2) muscular, or contractile tissue; (3) nervous, or conductile tissue; (4) fibrous, or connective tissue; (5) glandular, secreting or excreting tissue; (6) the blood, or nutrient tissue. The living part of all these is essentially formed by aggregations of cells—a cell being a small mass of matter spoken of as protoplasm, a soft jelly-like substance made up of an intricate combination of three gases, hydrogen, nitrogen, and oxygen, and of three solids, carbon, sulphur, and phosphorus.

1. Bony tissue.—This forms the solid framework of the body. It contains two parts intimately blended, one called the organic or animal, and the other the earthy or mineral substance. The organic part may be burnt away, leaving the mineral; and the earthy may be dissolved by acid, leaving the animal.

2. Muscular tissue consists of cells so differentiated as to be capable of considerable contractile power. There are three varieties of muscle: (a) Voluntary or striped muscle, (b) involuntary or unstriped muscle, (c) heart muscle.

3. Nervous tissue is composed of nerve-cells, special cells to receive, transform and originate impulses; and nerve-fibres, long threads of protoplasm to conduct sensations to the brain, and motor impulses to the muscles.

4. Connective tissue.—This takes the form chiefly of fibres, which serve to connect the various tissues together. There is white fibrous tissue and yellow elastic tissue. Fat or adipose tissue also comes under this heading.

5. Glandular tissue.—Glands are cells arranged in groups, which have the special function of separating useful material from the blood, or of getting rid of refuse matter from it.

6. The blood takes the part of the chief nutrient fluid of the body. It is composed of a liquid, the plasma, in which float corpuscles, or cells, of two kinds, the red, which are very numerous, and the white, which are much fewer in number.

In order to understand the anatomy of the body, we might compare the above tissues to the bricks, wood, iron, mortar, &c., which are employed in the building of a house, but when they have been placed properly together they will constitute different parts of the house, for instance, walls, floors, windows, &c.

In like manner in the human frame, or building, the organs are arranged in systems, and thus we have: (1) the osseous system, (2) the muscular system, (3) the digestive system, (4) the circulatory system, (5) the respiratory system, (6) the excretory system, (7) the absorptive system, (8) the nervous system. We shall in future lectures take up each of these systems in detail, and discuss at a later period the points of surgical interest in connection with them. It is hoped that in this way the "dry bones" may not only be covered with flesh, but with interest to all those desirous of knowing the elements of anatomy in conjunction with elementary surgery, especially in its bearing upon the nursing art.

### Addenbrooke's Hospital, Cambridge.

The quarterly meeting of the governors on the 31st ult. was adjourned for five weeks in order that the report of the committee on the accommodation for probationers and servants might be considered. At the meeting the Rev. E. G. Wood described the proposal to convert Addenbrooke's into a modern training school for nurses as "a leap in the dark," and said that to extend the training from two to three years, *i.e.*, to do justice to the paying probationers, was "a complete revolution." We have not the pleasure of Mr. Wood's acquaintance, but his utterances are calculated to cause surprise amongst intelligent hospital authorities all over the country. A month of the five weeks remains at the disposal of the governors, and we urge them individually to visit Addenbrooke's Hospital in that interval and go into the probationers' quarters, and the servants' quarters too. They will find that the servants have no ventilation whatever, in any proper meaning of that term, and that the probationers are no better off in this respect, whilst they are so crowded together that when all the cubicles in each room are occupied at the same time a few hours must render the atmosphere bad enough to be dangerous to the health of those who have to sleep in it.

They will further find that there is no accommodation for night nurses apart from the day nurses, and that every night nurse is liable to be aroused several times during her hours of rest owing to the necessity for the day staff to go in and out of the very place where she has to sleep. Of course governors must remember that on a bright sunshiny day rooms inhabited by ladies look outwardly bright and pleasant when unoccupied. Their hygienic condition can only be realised by experts or by the inexperienced who visit the rooms in question during the hours when they are in full occupation.

Speaking with the utmost deliberation, we beg to assure the governors of Addenbrooke's Hospital that unless they provide proper accommodation for their "probationers and servants," without a moment's delay, the credit of the whole institution may be sacrificed and the income at present derived from probationers' payments will deservedly and assuredly decrease.

### Dress and Uniforms.

By a Matron and Superintendent of Nurses.

### THE BROMPTON HOSPITAL.

This interesting little group represents a sister, staff-nurse, and probationer of the well-known Hospital for Consumption at Brompton. The sister, who occupies a chair in the centre, wears a neat dress of navy blue serge, the texture of which is so fine and smooth that, unlike most woollen materials, dust can have but little affinity with it. The skirt is made plain and full, and is turned up with a deep hem round the bottom. A tight-fitting bodice, with coat-shaped sleeves fastens in front, and is connected with the skirt by a band at the waist, which is the most suitable style for a uniform dress. The linen apron is hem-stitched, and is furnished with a bib that reaches nearly to the throat. Straps crossing at the back and fastening at the waist keep it in position. The cap is made of cambric, and when flat is of a triangular form. It is trimmed with two rows of goffered frilling, edged with narrow lace. The required shape is procured by means of a thread run through the material at the back, while another is taken along the reverse side of the gophers all round, to keep them in place. The degree of tightness with which these threads are drawn, is regulated by the size of the wearer's head. Strings of cambric, edged with lace, tie in a pretty little bow under the chin, and make a nice finish. Plain linen cuffs and collars are worn outside the dress, at the wrists and neck respectively.

The staff nurse wears a Galatea of original design—navy blue, shot with white, forms the groundwork, and this is relieved by three narrow white stripes close together, placed about half an inch apart. The skirt is full and plain, just clearing the ground, and is gathered into the band that holds the bodice. This latter is made tight to the figure and buttons in front. The apron is of linen, plainly hemmed, and has a high bib which fastens with straps that cross behind. The cap is circular, but drawn in somewhat full at the back, which makes it fit neatly to the head. Cambric is the material used, and it is finished off all round with a double row of goffered Coventry frilling. Linen cuffs and collar complete the costume.

Brompton Hospital Uniforms.

The probationer is attired in a pretty striped Galatea, consisting of three tiny pink stripes on a white ground. The effect is extremely fresh and cheerful, and it is, moreover, in perfect harmony with the beautiful and luxurious wards of the hospital. Her cap, apron, cuffs, and collars are in every respect the same as those worn by the staff nurse. The sisters' outdoor uniform is a Russian circular made of soft black Imperial cloth for summer, and of black Saxony for winter wear. The bonnet is a plain straw "princess" shape, trimmed with ribbon and velvet with strings of the same. The nurses and probationers wear a similar costume in navy blue

### Queen Victoria's Jubilee Institute.

DURING the past year 45 associations have become affiliated with the Queen's Jubilee Institute, 36 of them having been established in places where no district nursing association previously existed. The new branches are distributed as follows: England, 11; Rural Branch, 15; Scotland, 7; Ireland, 7; and Wales, 3. Certificates to the number of 98 have been received by Queen's nurses who have completed their term of agreement with the Institute. During the year 91 nurses completed their training in district nursing and are already settled at work in various parts of the country. Since the foundation of Queen Victoria's Jubilee Institute 523 Queen's Nurses have been placed on the roll, and Her Majesty has been pleased to approve the addition of the following: Superintendent: Charlotte E. Youngman, Manchester. Nurses: England—Helena Croft, Battersea; Sara Milroy, Chelsea; Frances A. Groves, Hammersmith; Lilian Ware, Manchester; Frances E. Mansell, East London; Alice Hardcastle, Manchester; Martha Beasley, Alice Buchanan, Catharine Thomas, Alice Wall, and Gertrude I. Quayle, Liverpool; Isabella I. Watson, Garston; Katharine M. Child, Edith Sayers, and Ethel Manley, Windsor; Sarah Kyle, Addlestone; Mary M. Taverner, Coventry; Bertha Bodley, Woolwich; Edith Mitchell and Eleanor L. Feast, Bath; Ellen Westcott and Agnes De Froissard, Torquay; Elizabeth Armstrong, Darlington; Mary Dole, Binbrook; Mary I. Ratliffe, Wisbech. Wales—Ada M. Magrath, Cardiff; E. R. Rutherford, Llandaff. Rural D. Branch—E. I. Beswick, Berrington; Kate Edwards, Bradford Peveril; M. H. Marshall, Burnham; K. D. Forest, Finedon; Lily Hames, Alton; A. E. Dagg, Gainsford. Ireland—Agnes Shaw, Aughrim; Alice Walshe, Dublin; Eileen Haugh and Amy Shannon, Londonderry; Kathleen Rogers, Dublin. Scotland—Agnes G. Small, B. I. Scott, and M. H. Thomson, Edinburgh; Isabella Jenkins, Aberlour; Janet Dickie, Pollockshaws; H. R. Maxwell, Larbert; H. B. Geikie, Dalbeattie; A. I. Warwick, Forfar; Janet White, Troon; S. E. Hutton, Lochwinnoch; M. H. Fleming, Blairgowrie; A. Mackenzie, Aberdeen; C. E. Copland and M. S. Frost, Kilmarnock.

THE RURAL DISTRICT BRANCH.

SIR,—Will you kindly allow us to call the attention of your readers to the work which our society is now doing in extending the nursing of the rural poor in their own homes by trained nurses?

We commenced the work five years ago under the title of the Rural Nursing Association. The Council of the Queen's Jubilee Institute, having assisted us greatly with both money and advice, have now incorporated us as a branch of the institute, we undertaking the rural, while they devote themselves to the urban, districts. The funds, however, remain quite distinct.

But, like every other association for charitable work, we are in want of money.

There is a great demand for our nurses; they are engaged as fast as they can be trained. There are plenty of candidates for training, who are able and willing to work, but who are not able to afford the cost of a really good nursing education. This is what we aim at giving them.

The district nursing is taught by various associations working in London and other large towns, and our probationers, while learning themselves, enable these associations to undertake the care of a greater number of poor patients than they otherwise could.

It is on these grounds, Sir, that we ask for money from those who are interested in the welfare of the poor both in town and country.

MAUD WOLMER.
LUCY C. HICKS-BEACH.
VICTORIA A. LAMBTON.
CHARLOTTE B. GREEN.

Rural District Branch of Queen Victoria's Jubilee Institute for Nurses, St. Katharine's Royal Hospital, Regent's Park, N.W.

### Presentations.

ON Christmas Day the nurses and probationers of Union Infirmary, Sunderland, presented Miss Cowan, their superintendent, with an afternoon tea table and copper tea kettle.

### Entertainments.

ON Saturday, December 29th, the annual Christmas tree entertainment for the children at the WESTERN INFIRMARY, Glasgow, took place. A number of subscribers and others interested in the hospital were present. After the tree was dismantled, tea was served in the conservatory. Dr. Finlayson addressed the nurses.

Christmas festivities began in the PUBLIC HOSPITAL AND DISPENSARY, Sheffield, on Sunday, December 23rd, at half-past two p.m., when the nurses, accompanied by the matron and house surgeons, made a tour through the wards, singing a selection of bright and pretty carols in each. They were assisted by several members of local choirs. The latter were absent on Christmas afternoon, when the carols were repeated, but one of the house surgeons and several ladies accompanied with cello and violins. A large number of the patients' friends were present on both occasions, and many of the patients joined in the singing. Entertainments were also organised on the 27th in all the wards, which were charmingly decorated, Christmas trees being provided in the children's wards.

A fresh proof of her interest in the VICTORIA HOSPITAL FOR CHILDREN, at Chelsea, was given by Princess Louise, Marchioness of Lorne, last week. Her Royal Highness, accompanied by the Marquis of Lorne and Lady Northesk, took part in the distribution of presents from the Christmas trees to the little patients. A large number of guests assembled on the occasion.

The annual entertainment at ST. JOHN'S HOSPITAL FOR SKIN DISEASE, in Leicester Square, took place on the 5th inst., at half-past five.

The concert for the patients at ST. MARY'S HOSPITAL, Paddington, was arranged for Wednesday, 9th inst., and the nurses' entertainment for the following evening.

A very successful reception was given at UNIVERSITY COLLEGE HOSPITAL on January 2nd, the decorations, the electric and other lights, the flowers, and the ferns quite transforming the buildings. This annual "patients' treat," always well attended, was this year considered more attractive than ever.

The inmates of the WESTMINSTER UNION WORKHOUSE had a highly amusing entertainment provided for them the other evening, some of the artistes from the Empire Theatre kindly giving their services on the occasion.

About fifty past and present patients received gifts from the handsome tree provided at the Christmas entertainment at WARMINSTER COTTAGE HOSPITAL. After the distribution had taken place an excellent tea was enjoyed, and this was followed by a concert. The wards were very prettily decorated, and amongst the visitors present were the Rev. Sir James and Lady Philipps, Lady Pelly, Mrs. Laverton, Mrs. Marten, the Misses Philipps, Dr. Wilcox, and Dr. Flower. An address was given by Sir James Philipps, who, in opening the meeting, expressed the regret with which they all anticipated the departure of the valued matron, Miss Wilks.

On Thursday, January 3rd, a concert was given at the DERBYSHIRE ROYAL INFIRMARY. The out-patient waiting-hall was very prettily decorated, and seated nearly 200 visitors and patients. A cantata for female voices was performed entirely by the nurses, the band being composed of residents, assisted by outside friends. The second part of the programme was a miscellaneous selection.

The Christmas festivities at the SAMARITAN FREE HOSPITAL FOR WOMEN AND CHILDREN were held on December 27th, and commenced with a musical and dramatic entertainment, after which the annual distribution of Christmas gifts took place, and, thanks to the liberality of a number of friends, many useful and ornamental presents were received by every patient, nurse, and servant in the hospital.

THE patients at the IVER, LANGLEY, and DENHAM COTTAGE HOSPITAL had an excellent dinner provided for them on Christmas and New Year's Days, and pleasant entertainments were also arranged by various friends. Nurse Elsby was congratulated on the decorations and on the excellent management which characterised the whole proceedings.

The annual Christmas entertainments of the Sunderland Union Hospital took place on December 26th. The entrance halls, wards, &c., were very tastefully decorated by the nurses. A tea was given to the male patients, followed by a concert, and on the Friday following the same entertainment was provided for the female patients. For the children a Christmas tree was dressed the next week.

At the Surbiton Cottage Hospital the Christmas decorations and entertainments were excellent. A fine tree laden with gifts was provided for the children, and many visitors, as well as the doctors and nursing staff, contributed to the amusement of the patients.

### Notes from India.

(Communicated.)

Lady Roberts' Home at Kasauli is closed at last after it has been practically empty for two years. It was an expensive venture, and said to be an unsuccessful one. Miss Sykes, lady superintendent of Lady Roberts' other home for sick officers, situate in the Murree Hills, has resigned and gone home, her place being taken by Deputy-Superintendent Harris, of the Indian Nursing Service, and she takes up the work in April. This arrangement will create a vacancy in the Indian Nursing Service, and give promotion to one of the nursing sisters. Miss Harris' station was at Peshawar; she was trained at St. Bartholomew's, came out to India in 1888, and was promoted to her present rank in October, 1890, and, with the exception of one year's furlough at home, has been ever since at Peshawar and the summer station of Cherat. The Murree Home has always been the most popular of Lady Roberts' Homes, and is now the last one left of those originally started. During the last year, it is reported that some 19 patients were treated there.

Miss A. S. Wildman has resigned the lady superintendentship of the Ramsay Hospital, Naini Tal. It is a paying hospital, and situated in one of the loveliest hill stations in India. The European portion has about 13 beds, and the charges for patients vary from 1s. 8d. to Rs. 8 per diem. The superintendent's duties are very similar to those of a matron at home, and she also has the training of probationers. Miss Wildman's departure is regretted, as she was very much liked and popular with her patients. The vacancy will probably be filled up before April, when the European portion of the hospital will be opened for the summer.

It is somewhat remarkable that the year's furloughs of all the deputy superintendents of the Indian nursing service will be due next trooping season; also the past senior of the nursing sisters. Unless some special arrangement is made, the junior nursing sisters will have not only to act for the deputy superintendents, but also to fill the important post of Lady Superintendent of Bombay. Probably there will be two vacancies this year in the Indian nursing service, and there is possibility of more next autumn.

The two gunners who were so badly hurt in the grand review at the Lahore Durbar are recovering, though their deaths have been several times reported. A great deal of sickness has followed the huge camp of Lahore, and there have been some deaths from pneumonia.

Nursing Sister Walters has been transferred from Allahabad to Quetta, and Sister Green from Quetta to Allahabad, and one or two changes are likely to take place in the Madras Presidency. The deputy superintendent at Rangoon has been working by herself for some time.

### Wants and Workers.

Can anyone recommend a home where a boy of weak intellect, aged ten years, will be received without payment? His mother is a poor widow, with six other children.

An old probationer asks if anyone can tell her where she could obtain a likeness of the late Miss A. Fisher?

The nurse who asked for a musical box for her patients in a lonely country infirmary writes in grateful acknowledgment of the success which followed her appeal made through our columns. A small organ was sent to her through the kind interest of another nurse.

### Training of Probationers at Lambeth Workhouse Infirmary.

The examinations of the probationers at Lambeth Workhouse Infirmary, which precede the distribution of certificates, will be conducted by an outside examiner, probably the medical superintendent of some other poor-law institution. Lectures for the Lambeth nurses were inaugurated long ago by Dr. Lloyd, who has done much to raise the status of the workers in the infirmary, where he has been so highly respected for nearly twenty years. Since the training school was formally organised two years ago, classes for the probationers have been regularly held by the superintendent of nurses, and the popularity of her instruction is proved by the fact that the staff nurses, with one single exception, asked to share in the second course. This request was complied with, the senior nurses being amongst the candidates for the impending examination, and they are to be congratulated on their laudable ambition to earn certificates. Lambeth School will attain a front rank by securing properly-conducted examinations as well as adequate instruction for its staff. It was recently proposed by some of the guardians to abolish the office of superintendent of nurses, but this would mean the destruction of the school, which obviously could not exist without a trained and certificated instructress. As a certificated trained nurse Miss Tilbury has done two years' excellent work with much tact, and has reason to be proud of its results, in the production of which she has received much assistance from the supervision and co-operation of so distinguished an officer as Dr. Lloyd.

### Where to Go.

New Hospital for Women, Euston Road, open from four to six p.m. on Thursday, January 24th.

Trained Nurses' Club, 12, Buckingham Street, Strand, on Friday evening, January 25th, sale of a few articles left over from the December sale of work held in aid of the club funds.

St. Thomas's Hospital.—The nurses' annual concert, Thursday, January 24th, at eight p.m.

St. James's Hall.—On Tuesday afternoon, January 22nd, a concert for the Invalid Children's Aid Association will be given, under the patronage of H.R.H the Duchess of York.

Bijou Theatre, Bayswater.—On Wednesday evening, January 15th, a fairy play will be performed by children, in aid of the funds of the Hospital for Sick Children, Great Ormond Street.

### Notes and Queries.

### Queries.

(55) *Nursing.*—Please recommend a book on the nursing of sick children (surgical) and on general ward management.—*Sister H. N.*

(56) *Uniform.*—Can anyone tell me whether the washing material worn by the Leeds district nurses is made specially for them?—*A Devonshire Nurse.*

(57) *Books.*—Please recommend a book on elementary anatomy.—*Nurse.*

(58) *Books.*—Could you recommend a book, not exceeding five shillings, on general nursing; also dictionary of diseases, with list of drugs?—*Sister A.*

### Answers.

(55) *Nursing (Sister H. N.)*—There is a chapter on this subject in Miss Lücke's "Lectures to Nurses"; and "Hospital Sisters," by same author, gives valuable hints on ward management. "A Text-book on Nursing," by Clara Weeks-Shaw, is very good, but dearer than the others. All can be obtained through the Scientific Press, 428, Strand. Also "Surgical Ward Work," by Dr. Miles. Did you read some articles on the "Nursing of Sick Children," in The Hospital of November 14th, 1891; January 23rd, 1892; February 6th, 27th; March 5th, 12th, 19th, 26th; April 16th?

(56) *Uniform (A Devonshire Nurse).*—If you write direct to Leeds you will doubtless receive a prompt and courteous reply.

(57) *Books (Nurse).*—In Dr. Lawrence Humphry's "Manual of Nursing; Medical and Surgical," there is an excellent chapter, illustrated, on this subject, and a course of articles, commencing this week in The Hospital, will probably prove to be exactly the form of instruction you wish for.

(58) *General Nursing (Sister A.).*—"Helps in Sickness," Scientific Press.—You cannot get all you want in one volume. Hoblyn's Dictionary Medical Terms, or Quain's Dictionary of Medicine, or Materia Medica would help you, but they are expensive. Write for catalogue to Scientific Press, 428, Strand, and read answers given to other correspondent.

## Figures and Tables

**Figure f1:**